# It is safe to use transdermal glyceryl trinitrate to lower blood pressure in patients with acute ischaemic stroke with carotid stenosis

**DOI:** 10.1136/svn-2019-000232

**Published:** 2019-03-19

**Authors:** Jason P Appleton, Lisa J Woodhouse, Andrew Belcher, Daniel Bereczki, Eivind Berge, Valeria Caso, Hui Meng Chang, Hanne K Christensen, Ronan Collins, John Gommans, Ann C Laska, George Ntaios, Serefnur Ozturk, Gillian M Sare, Szabolcs Szatmari, Yongjun Wang, Joanna M Wardlaw, Nikola Sprigg, Philip M Bath

**Affiliations:** 1 Stroke, Division of Clinical Neurosciences, University of Nottingham, Nottingham, UK; 2 Stroke, Nottingham University Hospitals NHS Trust, Nottingham, UK; 3 Department of Neurology, Semmelweis University, Budapest, Hungary; 4 Department of Internal Medicine and Cardiology, Oslo University Hospital, Oslo, Norway; 5 Stroke Unit, Santa Maria della Misericordia Hospital, University of Perugia, Perugia, Italy; 6 Department of Neurology, Singapore General Hospital, Singapore, Singapore; 7 Neurology, Bispebjerg and Frederiksberg Hospital, Copenhagen, Denmark; 8 Tallaght Hospital, Trinity College Dublin, Dublin, Ireland; 9 Department of Medicine, Hawke’s Bay District Health Board, Hastings, New Zealand; 10 Department of Clinical Science, Danderyd Hospital, Karolinska Institute, Stockholm, Sweden; 11 Department of Medicine, University of Thessaly, Larissa, Greece; 12 Neurology, Selcuk University Faculty of Medicine, Konya, Turkey; 13 Neurology, Nottingham University Hospitals NHS Trust, Nottingham, UK; 14 Department of Neurology, Clinical County Emergency Hospital, Targu Mures, Romania; 15 Neurology, Beijing Tiantan Hospital, Beijing, China; 16 Centre for Clinical Brain Sciences, Edinburgh, UK

**Keywords:** acute stroke, carotid stenosis, glyceryl trinitrate, blood pressure, safety

## Abstract

**Background:**

There is concern that blood pressure (BP) lowering in acute stroke may compromise cerebral perfusion and worsen outcome in the presence of carotid stenosis. We assessed the effect of glyceryl trinitrate (GTN) in patients with carotid stenosis using data from the Efficacy of Nitric Oxide in Stroke (ENOS) Trial.

**Methods:**

ENOS randomised 4011 patients with acute stroke and raised systolic BP (140–220 mm Hg) to transdermal GTN or no GTN within 48 hours of onset. Those on prestroke antihypertensives were also randomised to stop or continue their medication for 7 days. The primary outcome was the modified Rankin Scale (mRS) at day 90. Ipsilateral carotid stenosis was split: <30%; 30–<50%; 50–<70%; ≥70%. Data are ORs with 95% CIs adjusted for baseline prognostic factors.

**Results:**

2023 (60.5%) ischaemic stroke participants had carotid imaging. As compared with <30%, ≥70% ipsilateral stenosis was associated with an unfavourable shift in mRS (worse outcome) at 90 days (OR 1.88, 95% CI 1.44 to 2.44, p<0.001). Those with ≥70% stenosis who received GTN versus no GTN had a favourable shift in mRS (OR 0.56, 95% CI 0.34 to 0.93, p=0.024). In those with 50–<70% stenosis, continuing versus stopping prestroke antihypertensives was associated with worse disability, mood, quality of life and cognition at 90 days. Clinical outcomes did not differ across bilateral stenosis groups.

**Conclusions:**

Following ischaemic stroke, severe ipsilateral carotid stenosis is associated with worse functional outcome at 90 days. GTN appears safe in ipsilateral or bilateral carotid stenosis, and might improve outcome in severe ipsilateral carotid stenosis.

## Introduction

Blood pressure (BP) is elevated in 75% of patients presenting with acute ischaemic stroke^1^ and is associated independently with poor clinical outcomes.^2 3^ Lowering elevated BP appears safe in acute ischaemic stroke, but has failed to show clinical benefit.^4^ There is a specific concern regarding BP lowering in the 15% of patients with significant carotid stenosis in whom cerebral perfusion may be compromised and where reducing BP might extend the ischaemic core and potentially worsen outcome.^5^ Data on BP reduction in severe ipsilateral or bilateral carotid stenosis are limited, although a meta-analysis found that lower BP was associated with an increased rate of stroke recurrence in bilateral carotid stenosis.^6^ In the ultra-acute prehospital and acute hospital situation, information on carotid stenosis is often not available and it is unclear whether BP lowering is safe in this group of patients with stroke.

The Efficacy of Nitric Oxide in Stroke (ENOS) Trial assessed the safety and efficacy of transdermal glyceryl trinitrate (GTN) and of continuing prestroke antihypertensives in 4011 patients with acute stroke.^7^ Although GTN lowered BP by 7/3.5 mm Hg at day 1, GTN did not influence functional outcome at 90 days.^7^ However, when administered within 6 hours of stroke onset GTN improved several clinical outcomes.^8^ The aim of the current preplanned substudy^9^ was to assess the safety and efficacy of BP lowering on clinical outcomes in patients with acute ischaemic stroke and carotid stenosis.

## Methods

Details pertaining to the ENOS trial protocol, statistical analysis plan, baseline characteristics and main trial results have been published.^8 10–12^ In summary, the ENOS Trial recruited 4011 patients with acute stroke within 48 hours of onset with high systolic BP (140–220 mm Hg) and randomised them to GTN 5 mg patch or no patch for 7 days. Those participants taking antihypertensive medication prior to their index event were also randomised to continue or stop these drugs for 7 days. Known carotid stenosis was not an exclusion criterion. Written consent to participate was given by patients or relatives/carers in those who lacked capacity. The ENOS Trial was registered (ISRCTN99414122).

### Patient and public involvement

Patients and public were not involved in the development of this preplanned secondary analysis of the ENOS trial.

### Carotid stenosis

Clinical information on carotid stenosis was collected by investigators during the participant’s index event admission. Clinical imaging using either carotid Doppler, MR angiography or CT angiography was performed as per local protocol. Investigators entered the % of stenosis of both left and right internal carotid arteries using North American Symptomatic Carotid Endarterectomy Trial (NASCET) criteria where available (online supplementary material).^13^ Data were checked and validated but no central adjudication of carotid imaging was performed.

10.1136/svn-2019-000232.supp1 Supplementary data

Participants who had a final diagnosis of ischaemic stroke and who had carotid data available were included in this substudy. Grades of carotid stenosis were defined as follows:

- Unilateral carotid stenosis ipsilateral to the symptomatic hemisphere:^13^ <30%, 30–<50%, 50–<70%, ≥70%.
- Bilateral carotid stenosis (% for both carotid arteries): <30%, 30–<50%, ≥50%.

### Haemodynamic measures

BP and heart rate were measured peripherally at baseline (three measurements) and on days 1–7 (two measurements/day), using validated automated equipment (Omron 705 CP).^14^

### Clinical outcomes

The primary outcome in ENOS was functional outcome measured using the modified Rankin Scale^15^ (mRS, a seven-level categorical scale where 0=independent and 6=dead) at 90 days. Day 90 secondary outcomes included disability (Barthel Index),^16^ mood (Zung Depression Scale),^17^ quality of life (health utility status calculated using the European Quality of Life 5-dimensions three levels version, and Visual Analogue Scale)^18^ and cognition (telephone mini-mental state examination,^19^ modified Telephone Interview for Cognition Scale [TICS-M]^20^ and verbal fluency). Patients who had died by day 90 were assigned a worst score for the outcomes. Safety data were collected on all-cause mortality at day 90, early neurological deterioration (a minimum 5-point reduction overall or >2 points reduction in the consciousness domain from baseline to day 7 on the Scandinavian Stroke Scale [SSS]), symptomatic hypotension, hypertension or headache by day 7. The National Institute of Health Stroke Scale (NIHSS) was calculated from SSS.^21^ Day 90 outcomes were recorded by trained blinded assessors via telephone at national coordinating centres.

### Statistical analysis

In line with the ENOS Trial statistical analysis plan and statistical analyses performed in the primary publication, intention-to-treat analysis of data was carried out.^11^ Data are number (%), median [IQR] or mean (SD). Baseline characteristics between grades of carotid stenoses were assessed using the χ^2^test for categorical variables and one-way analysis of variance for continuous variables.

Associations between carotid stenosis grades and outcomes were assessed using multiple linear regression, ordinal logistic regression or binary logistic regression with adjustment for baseline prognostic covariates including age, sex, baseline mRS score, history of previous stroke, history of diabetes mellitus, total anterior circulation stroke, nitrate use, baseline SSS, thrombolysis, feeding status, time to randomisation and baseline systolic BP. Analyses involving the whole population were also adjusted for treatment allocation. Associations between BP change from baseline to day 1 and outcome across degrees of carotid stenosis were assessed per 10 mm Hg reduction in BP. Interaction p values were obtained by adding an interaction term to statistical models. Data are mean difference or OR and associated 95% CIs, with significance defined as p≤0.05. Analyses were performed using SPSS V.24 (Chicago, Illinois, USA).

## Results

Of 4011 participants, 2023 (50.4%) had a final diagnosis of ischaemic stroke and carotid imaging data (GTN 1002 vs no GTN 1021, table 1). One thousand three hundred and nineteen (32.9%) patients with ischaemic stroke did not have carotid imaging, typically those with more severe stroke (carotid imaging: SSS 36.6 [12.4], no imaging: SSS 30.6 [13.7], p<0.001); there was no relationship between country of enrolment and whether carotid imaging was performed (data not shown). Of 2023 participants with carotid data, 148 (7.3%) had 50–<70% ipsilateral stenosis, 213 (10.5%) had ≥70% ipsilateral stenosis and 97 (4.8%) had ≥50% bilateral stenosis. Age and rates of treated hypertension, diabetes mellitus, atrial fibrillation, history of transient ischaemic attack, ischaemic heart disease and peripheral arterial disease differed across grades of ipsilateral carotid stenosis. Those with higher degrees of ipsilateral carotid stenosis were more likely to be male, current smokers, have more severe strokes with higher NIHSS and lower Glasgow Coma Scale Scores, fewer cardioembolic and small vessel disease-related strokes, and more received thrombolysis treatment (table 1). As compared with patients with carotid imaging data, those without had a worse functional outcome at day 90: mRS 4 [3] versus 3 [3], (OR 1.76, 95% CI 1.54 to 2.01, p<0.001).

**Table 1:** Baseline characteristics of all patients with ischaemic stroke with carotid data and by ipsilateral carotid stenosis

	All IS	GTN	No GTN	Continue	Stop	Stenosis <30%	Stenosis 30–<50%	Stenosis 50–<70%	Stenosis ≥70%	P value
Number of patients	2023	1002	1021	534	525	1431	224	148	213	
Age (years)	69.1 (11.4)	68.8 (11.3)	69.4 (11.5)	71.6 (10.5)	70.9 (10.5)	68.3 (11.6)	71.2 (10.7)	73.3 (9.9)	68.9 (10.8)	<0.001
Sex, male (%)	1193 (59.0)	599 (59.8)	594 (58.2)	286 (53.6)	283 (53.9)	817 (57.1)	141 (62.9)	91 (61.5)	141 (66.2)	0.036
Premorbid mRS>1 (%)	209 (10.3)	95 (9.5)	114 (11.2)	71 (13.3)	64 (12.2)	135 (9.4)	27 (12.1)	20 (13.5)	24 (11.3)	0.29
Medical history (%)										
Hypertension	1307 (64.6)	624 (47.7)	683 (66.9)	512 (95.9)	503 (95.8)	903 (63.1)	146 (65.2)	108 (73.0)	144 (67.6)	0.078
Treated hypertension	1072 (53.0)	516 (51.5)	556 (54.5)	533 (99.8)	522 (99.4)	720 (50.3)	134 (59.8)	98 (66.2)	115 (54.0)	<0.001
Diabetes mellitus	353 (17.4)	164 (16.4)	189 (18.5)	125 (23.4)	121 (23.0)	245 (17.1)	38 (17.0)	38 (25.7)	28 (13.1)	0.020
Atrial fibrillation	333 (16.5)	169 (16.9)	164 (16.1)	135 (25.3)	116 (22.1)	224 (15.7)	43 (19.2)	36 (24.3)	30 (14.1)	0.024
Stroke	295 (14.6)	150 (15.0)	145 (14.2)	113 (21.2)	97 (18.5)	207 (14.5)	37 (16.5)	25 (16.9)	22 (10.3)	0.22
TIA	286 (14.1)	147 (14.7)	139 (13.6)	91 (17.0)	96 (18.3)	179 (12.5)	43 (19.2)	24 (16.2)	39 (18.3)	0.010
IHD	380 (18.8)	191 (19.1)	189 (18.5)	136 (25.5)	153 (29.1)	248 (17.3)	59 (26.3)	38 (25.7)	34 (16.0)	0.001
PAD	65 (3.2)	29 (2.9)	36 (3.5)	23 (4.3)	22 (4.2)	35 (2.4)	10 (4.5)	8 (5.4)	12 (5.6)	0.018
Hyperlipidaemia	587 (29.0)	293 (29.2)	294 (28.8)	204 (38.2)	216 (41.1)	412 (28.8)	60 (26.8)	52 (35.1)	57 (26.8)	0.29
Smoking, current	573 (28.3)	278 (27.7)	295 (28.9)	111 (20.8)	109 (20.8)	380 (26.6)	71 (31.7)	37 (25.0)	83 (39.0)	0.010
Alcohol >21 units per week	176 (8.7)	92 (9.2)	84 (8.2)	38 (7.1)	29 (5.5)	116 (8.1)	19 (10.9)	11 (7.4)	28 (13.1)	0.10
Side of lesion, right (%)	1047 (51.8)	509 (50.8)	538 (52.7)	278 (52.4)	264 (50.4)	722 (50.5)	109 (48.7)	89 (60.1)	127 (59.6)	0.010
NIHSS (/42), calculated	9.9 (5.3)	9.8 (5.3)	10.0 (5.4)	10.3 (5.5)	10.2 (5.4)	9.7 (5.2)	10.1 (5.4)	9.6 (5.0)	11.6 (5.6)	<0.001
GCS <15 (%)	460 (22.7)	222 (22.2)	238 (23.3)	134 (25.1)	145 (27.6)	300 (21.0)	61 (27.2)	32 (21.6)	65 (30.5)	0.006
TOAST classification*										
Cardioembolic	358 (17.7)	181 (18.1)	177 (17.3)	133 (24.9)	117 (22.3)	271 (18.9)	41 (18.3)	24 (16.2)	22 (10.3)	0.021
Large vessel	527 (26.1)	254 (25.3)	273 (26.7)	143 (49.5)	146 (27.8)	200 (14.0)	53 (23.7)	90 (60.8)	180 (84.5)	<0.001
Small Vessel	808 (39.9)	402 (40.1)	406 (39.8)	188 (35.2)	199 (37.9)	649 (45.4)	105 (46.9)	34 (23.0)	16 (7.5)	<0.001
Other	394 (19.5)	202 (20.2)	192 (18.8)	93 (17.4)	87 (16.6)	333 (23.3)	32 (14.3)	17 (11.5)	12 (5.6)	<0.001
Haemodynamics										
BP, systolic (mm Hg)	166.6 (18.5)	167.1 (18.3)	166.1 (18.7)	165.4 (18.9)	167.7 (17.8)	166.6 (18.6)	166.6 (18.7)	165.1 (17.2)	167.5 (18.3)	0.70
BP, diastolic (mm Hg)	89.2 (13.0)	89.9 (13.1)	88.5 (12.8)	87.5 (13.3)	88.2 (12.6)	90.1 (13.0)	88.2 (13.3)	87.7 (12.9)	86.7 (11.8)	<0.001
Heart rate (bpm)	76.8 (14.4)	77.1 (14.5)	76.5 (14.2)	75.8 (14.5)	76.1 (14.5)	76.5 (14.6)	78.4 (13.1)	78.7 (15.4)	75.3 (13.1)	0.039
Time to randomisation [hours]	25.6 [21.2]	24.9 [21.5]	26.0 [21.1]	25.2 [18.8]	23.9 [22.1]	26.0 [21.6]	24.0 [20.1]	23.9 [17.8]	24.8 [19.0]	0.08
Thrombolysis (%)	239 (11.8)	107 (10.7)	132 (12.9)	71 (13.3)	63 (12.0)	156 (10.9)	24 (10.7)	20 (13.5)	39 (18.3)	0.015

*Total may exceed 100% due to mixed causality. χ^2^ test for categorical variables or one-way analysis of variance for continuous variables across grades of carotid stenosis.

BP, blood pressure; GCS, Glasgow Coma Scale; GTN, glyceryl trinitrate; IHD, ischaemic heart disease; mRS, modified Rankin Scale; NIHSS, National Institute of Health Stroke Scale; PAD, peripheral arterial disease; TIA, transient ischaemic attack.

### Relationship between carotid stenosis and outcome

**Figure 1:**
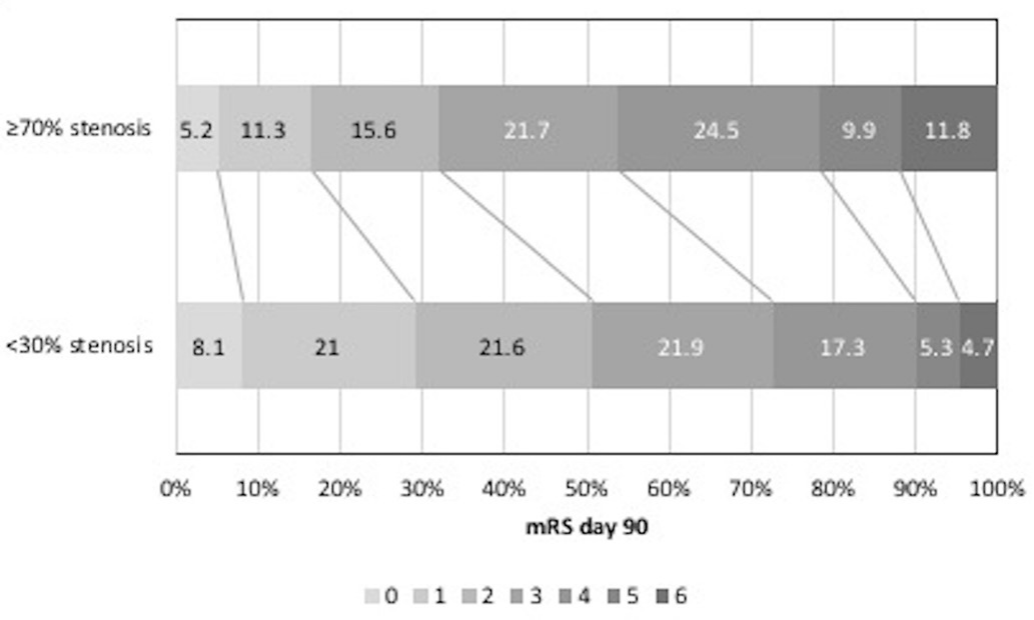
mRS at day 90 <30% vs ≥70% ipsilateral stenosis. mRS, modified Rankin Scale.

Across all patients and as compared with participants with <30% ipsilateral stenosis, those with ≥70% stenosis had an unfavourable shift in mRS (worse outcome) at day 90 (OR 1.88, 95% CI 1.44 to 2.44, p<0.001, table 2, figure 1); significant associations with worse disability and quality of life, more depression and poorer cognitive scores were also seen. In addition, those with ≥70% stenosis had an increased rate of recurrent ischaemic stroke, clinical deterioration, neurological deterioration, and higher NIHSS Scores at day 7 (online supplementary table 1).

**Table 2:** Functional outcome and death at day 90 by degree of ipsilateral carotid stenosis

	Stenosis <30%	Stenosis 30–<50%			Stenosis 50–<70%			Stenosis ≥70%		
		n (%) /median [IQR]	OR (95% CI)	P value	n (%) /median [IQR]	OR (95% CI)	P value	n (%) /median [IQR]	OR (95% CI)	P value
Number of participants	1431	224	–	–	148	–	–	213	–	–
mRS (/6)*	2 [3]	2 [2]	1.03 (0.80 to 1.33)	0.83	3 [2]	1.21 (0.89 to 1.64)	0.23	3 [2]	1.88 (1.44 to 2.44)	<0.001
Death (%)	67 (4.7)	21 (9.4)	1.85 (1.06 to 3.22)	0.030	11 (7.4)	1.43 (0.71 to 2.90)	0.32	25 (11.8)	2.52 (1.48 to 4.27)	0.001

Data are n (%), median (IQR) or OR with 95% CIs. Comparison using logistic or ordinal regression with <30% stenosis as reference group. Adjusted for age, sex, baseline mRS, history of previous stroke, history of diabetes mellitus, TACS, nitrate use, baseline SSS, thrombolysis, feeding status, time to randomisation, baseline SBP, GTN/no GTN and continue/stop.

*Ordinal logistic regression.

GTN, glyceryl trinitrate; mRS, modified Rankin Scale; SSS, Scandinavian Stroke Scale.

### Effects of GTN versus no GTN

**Figure 2:**
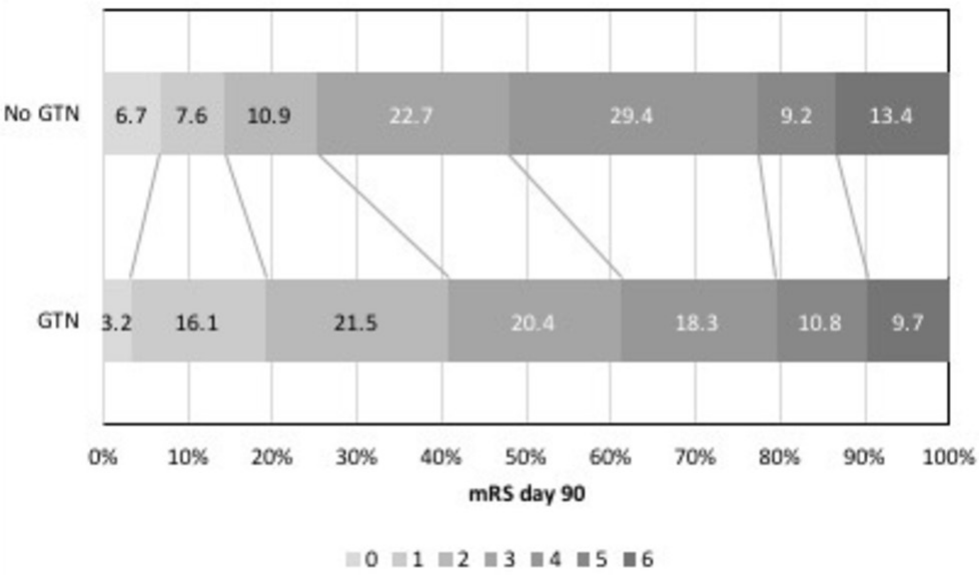
mRS at day 90 in those with ≥70% ipsilateral stenosis GTN versus no GTN. GTN, glyceryl trinitrate; mRS, modified Rankin Scale.

Those with ≥70% ipsilateral stenosis who were randomised to GTN had a significant shift in mRS to less death or dependency at 90 days (OR 0.56, 95% CI 0.34 to 0.93, p=0.024) (table 3, figure 2). However, GTN did not influence mRS across the other carotid stenosis groups, although there were higher cognitive scores at day 90 in those with 50–<70% stenosis but not in other stenosis groups. Headache, a recognised side effect of GTN, was more common in those with ≥70% stenosis who were randomised to GTN; non-significant increases in headache with GTN were also reported in the other carotid stenosis groups (online supplementary table 2).

**Table 3:** Functional outcome and death at day 90 by randomised treatment by degree of ipsilateral carotid stenosis

	Stenosis 30–<50%				Stenosis 50–<70%				Stenosis ≥70%			
	GTN	No GTN	OR (95% CI)	P value	GTN	No GTN	OR (95% CI)	P value	GTN	No GTN	OR (95% CI)	P value
Number of participants	102	122	–	–	77	71	–	–	94	119	–	–
mRS (/6)*	2 [3]	3 [2]	0.77 (0.47 to 1.27)	0.31	3 [2]	3 [2]	0.71 (0.39 to 1.31)	0.28	3 [2]	4 [2]	0.56 (0.34 to 0.93)	0.024
Death (%)	7 (6.9)	14 (11.5)	0.43 (0.14 to 1.32)	0.14	3 (3.9)	8 (11.3)	0.18 (0.03 to 1.03)	0.054	9 (9.7)	16 (13.4)	0.63 (0.23 to 1.75)	0.37

	Continue	Stop			Continue	Stop			Continue	Stop		
Number of participants	65	68	–	–	52	47	–	–	57	57	–	–
mRS (/6)*	2 [3]	2.5 [3]	0.93 (0.48 to 1.80)	0.82	3 [2]	3 [2]	1.92 (0.86 to 4.27)	0.11	4 [3]	4 [3]	1.46 (0.73 to 2.93)	0.29
Death (%)	9 (13.8)	7 (10.3)	1.59 (0.42 to 6.01)	0.49	6 (11.5)	1 (2.1)	81.00 (1.08 to 6093.98)	0.046	12 (21.4)	6 (10.5)	3.11 (0.85 to 11.44)	0.09

Data are n (%), median (IQR) or OR with 95% CIs. Comparison using logistic or ordinal regression. Adjusted for age, sex, baseline mRS, history of previous stroke, history of diabetes mellitus, TACS, nitrate use, baseline SSS, thrombolysis, feeding status, time to randomisation, baseline SBP and continue/stop or GTN/no GTN, respectively.

*Ordinal logistic regression.

GTN, glyceryl trinitrate; mRS, modified Rankin Scale; SBP, systolic blood pressure; SSS, Scandinavian Stroke Scale; TACS, total anterior circulation syndrome.

### Effects of continuing versus stopping prestroke antihypertensives

In those with 50–<70% ipsilateral stenosis, continuing prestroke antihypertensives was associated with more depression, worse disability and quality of life, and poorer cognitive score (TICS-M) at day 90 compared with stopping prestroke antihypertensives, independent of GTN allocation. No effect on mRS was seen nor were these effects replicated in those with more severe (≥70%) ipsilateral stenosis (table 3).

### Bilateral carotid stenosis

Only 97/2023 (4.8%) had ≥50% bilateral carotid stenosis. There were no significant associations across degrees of bilateral carotid stenosis with clinical outcome measures at either 7 or 90 days (online supplementary table 4). Neither GTN nor continuing prestroke antihypertensives influenced outcome in participants with bilateral carotid stenosis (data not shown).

### BP change and carotid stenosis

The largest fall in BP was seen from baseline to day 1 in those randomised to GTN versus no GTN (7/3 mm Hg overall) and did not significantly differ across degrees of ipsilateral (online supplementary table 2, interaction p=0.22) or bilateral carotid stenosis (interaction p=0.19). When assessed across degrees of ipsilateral carotid stenosis, change in BP from baseline to day 1 did not influence mRS at day 90 (online supplementary table 5). There were no significant interactions between treatment with GTN or continuing prestroke antihypertensives in relation to BP change and outcome. Similarly, across degrees of bilateral carotid stenosis, change in BP from baseline to day 1 did not influence mRS at day 90 (online supplementary table 6).

## Discussion

In this preplanned ENOS substudy,^9^ taking all patients irrespective of treatment allocation, the presence of severe (≥70%) ipsilateral symptomatic carotid stenosis was associated with unfavourable clinical outcomes both early and late after acute stroke. However, treatment with GTN versus no GTN was associated with a significant shift to less death or dependency at 90 days in those participants with ≥70% ipsilateral stenosis. Continuing versus stopping prestroke antihypertensives was associated with worse secondary outcomes in those with 50–<70% ipsilateral stenosis. Modest BP lowering was safe in patients with acute stroke in the context of unilateral and bilateral carotid stenosis.

It has long been established that higher degrees of symptomatic carotid stenosis are associated with early stroke recurrence and subsequent dependency after minor stroke and transient ischaemic attack; carotid endarterectomy is therefore recommended.^22^ This dataset suggests that patients with more severe strokes with large artery disease who may not have been eligible for carotid revascularisation have both poor early and late clinical outcomes as expected. These findings are likely to be even stronger in clinical practice as the 32.9% patients with ischaemic stroke who did not have carotid imaging had more severe strokes and therefore worse clinical outcome than those with imaging.

Although pathophysiological data have demonstrated dysfunctional cerebral autoregulation in severe carotid stenosis,^23–25^ and cerebral blood flow can become dependent on systemic BP,^5^ there are limited prospective data assessing BP lowering in acute stroke with carotid stenosis. Previous blinded analysis of ENOS performed during recruitment revealed that BP lowering in the context of carotid stenosis was safe.^9^ We have reinforced that finding here with no evidence to suggest modest BP lowering is associated with stroke recurrence or other poor outcomes. A post hoc analysis of the Scandinavian Candesartan Acute Stroke Trial (SCAST) found no clear evidence to suggest that BP lowering was detrimental in patients with carotid stenosis, but there were non-significant tendencies towards increased stroke progression and poor functional outcome with candesartan.^26^ As in the present substudy, the observed BP lowering effect in SCAST was modest (5/2 mm Hg) and therefore differing drug class effects may explain the differences observed between the present analysis and SCAST. Further, continuing prestroke antihypertensives in those with modest ipsilateral carotid stenosis (50–<70%) was associated with poorer outcomes across several secondary domains, but not in those with ≥70% ipsilateral stenosis. This may be due to those with modest stenosis having greater baseline comorbidity (higher mRS, more hypertension, diabetes, previous stroke, ischaemic heart disease and hyperlipidaemia) than those with ≥70% stenosis. This imbalance may represent selection bias, whereby patients with more severe stroke and severe stenosis were not imaged as doing so would not change management. It is unclear whether there are drug class-specific mechanisms that may be harmful in the context of carotid stenosis early after stroke, but we add to evidence from the main ENOS Trial that routinely continuing prestroke antihypertensives should perhaps be avoided until the patient is neurologically stable.^7^ Whether larger precipitous drops in BP are safe is beyond the scope of this substudy, but given the uncertainty this practice should perhaps be avoided.

The shift in mRS to less death or dependency with GTN seen in those with ≥70% ipsilateral carotid stenosis may be related to effects other than BP lowering. As a vasodilator, GTN did not reduce cerebral blood flow in patients with acute stroke despite lowering peripheral and central BP.^27 28^ Although it is unclear whether GTN improves cerebral blood flow,^28^ it can be hypothesised that GTN may improve collateral blood supply via surface pial collaterals^29^ and thereby maintain blood flow to the ischaemic penumbra in the context of carotid stenosis when given early. A similar potential beneficial effect of transdermal GTN administered within 4 hours of stroke onset in patients with ≥70% ipsilateral carotid stenosis was seen in a preliminary analysis of 314 patients from the Rapid Intervention with Glyceryl trinitrate in Hypertensive stroke Trial-2 (RIGHT-2).^30^

Bilateral carotid stenosis is uncommon (4.8% in this analysis) and therefore data regarding the safety of BP lowering in patients with acute stroke in this context are scanty.^6^ Impaired cerebral perfusion is more common in this population than in unilateral carotid stenosis and although we found no evidence that modest BP lowering was unsafe, further data are required to address this question.

This study represents the largest trial-based analysis of BP lowering in patients with acute ischaemic stroke with carotid stenosis to date. However, there are limitations. First, not all patients with ischaemic stroke had carotid imaging studies performed, a deficiency most prominent in patients with severe stroke who would not be appropriate candidates for revascularisation therapy. Second, the analyses across degrees of carotid stenosis are subgroup analyses and, so the results may represent chance. Third, the median time to randomisation in ENOS was 26 hours and so the effect of BP lowering in the context of carotid stenosis within the first few hours of ischaemic stroke remains unclear. Fourth, no adjustment was made for multiplicity of testing due to the exploratory nature of the study. Fifth, imaging information on carotid stenosis was provided by investigators at the site with unknown reporting criteria (NASCET,^13^ European Carotid Surgery Trial [ECST]),^31^ and Carotid and Vertebral Artery Transluminal Angioplasty Study (CAVATAS)^32^) and were not centrally adjudicated. However, data were validated and checked for accuracy. Sixth, it was not possible to adjust cognitive outcome data for cognition at baseline and many of the differences seen at 3 months in association with degrees of stenosis (apart from allocated trial treatment differences) likely reflect baseline status. Seventh, ENOS assessed mild to moderate BP lowering and the results presented here do not provide information on the effects of intensive BP lowering. Last, data on carotid endarterectomy were not captured prospectively in ENOS and were therefore unavailable for the population studied, although in a population of patients with mostly moderate-to-severe stroke it is unlikely that many patients underwent endarterectomy.

In summary, this ENOS substudy has demonstrated that severe ipsilateral carotid stenosis is associated with worse clinical outcomes at 7 days and 90 days after acute stroke irrespective of treatment allocation. Transdermal GTN improved functional outcome at 90 days in those with ≥70% ipsilateral stenosis and was safe across all degrees of carotid stenosis whether unilateral or bilateral. Further, modest BP lowering with GTN was safe in the context of carotid stenosis although continuing prestroke antihypertensives was associated with poorer secondary outcomes in those with 50–<70% ipsilateral carotid stenosis. Future studies should establish whether GTN and other BP lowering therapies have specific mechanistic properties that may be of benefit in acute stroke in the context of carotid stenosis.
