# Supplementary material for: It is safe to use transdermal glyceryl trinitrate to lower blood pressure in patients with acute ischaemic stroke with carotid stenosis
Source: Stroke Vasc Neurol. 2019 Mar 19;4(1):28–35. doi: 10.1136/svn-2019-000232 (PMC6475087; doi:10.1136/svn-2019-000232)
Supplement: Supplementary data [file svn-2019-000232supp001.pdf]

Supplementary Material for: Blood pressure lowering using transdermal glyceryl trinitrate in acute ischaemic stroke patients with carotid stenosis is safe

1. Efficacy of Nitric Oxide in Stroke (ENOS) trial steering and international advisory committees
2. ENOS carotid stenosis working practice document
3. Supplementary tables

1. **Efficacy of Nitric Oxide in Stroke (ENOS) trial steering and international advisory committees**

Trial Steering Committee:
D Thomas (Independent Chair to 2006, UK), G Venables (Independent Chair from 2006, UK), P Amarenco (Independent Physician, France), K Muir (Independent Physician, UK), P M W Bath (Chief Investigator, UK), N Sprigg (Deputy Chief Investigator, UK), E Berge (Norway), K R Lees (UK), S Pocock (Statistician from 2003, UK), A Shone (Sponsor's Representative, UK), A Skene (Statistician to 2003, UK), J M Wardlaw (Neuroradiologist, UK), D Whynes (Health economist, UK).

International Advisory Committee:
P M W Bath (Chair, UK), E Berge (Norway), M Beridze (Georgia), C Bladin (Australia), V Caso (Italy), C Chen/H M Chang (Singapore), H Christensen (Denmark), R Collins (Eire), A Czlonkowska (Poland), E Díez-Tejedor (Spain), A El Etribi (Egypt), A R Ghani (Malaysia), J Gommans (New Zealand), A C Laska (Sweden), K R Lees (UK), J Navarro (Philippines), G Ntaios (Greece), S Ozturk (Turkey), S Phillips (Canada), K Prasad (India), H A de Silva (Sri Lanka), S Szatmari (Romania), L Wong (Hong Kong), Y-J Wang (China).

2. ENOS carotid stenosis working practice document

ENOS TRIAL

INTERNAL CAROTID ARTERY STENOSIS (ICA)

Completion of Hospital Event Information

PEAK SYSTOLIC VELOCITY (PSV)		% DIAMETER REDUCTION		ENTER ON HOSPITAL EVENT FORM Equivalent mid-point
1-109		0-29		14
110-130		30-49		39
>130		50-69		59
>210		70-95		82
"STRING FLOW"		96-99		98
"NO FLOW"		100		100

<u>Both RIGHT and LEFT ICA needs entering</u>

If the report states:

An exact percentage, then enter as reported

Between, or less than a certain percentage i.e. 0-29 or less than 29%, then enter the mid-point i.e. 14

If the report gives a velocity then find the equivalent mid-point percentage and enter this.

3. Supplementary tables

Supplementary table 1: Secondary outcomes by degree of ipsilateral carotid stenosis

	Stenosis <30%	Stenosis 30-<50%			Stenosis 50-<70%			Stenosis ≥70%		
		n (%) / mean (SD)	OR/MD (95% CI)	p	n (%) / mean (SD)	OR/MD (95% CI)	p	n (%) / mean (SD)	OR/MD (95% CI)	p
Number of Participants	1431	224	-	-	148	-	-	213	-	-
Day 7 (or discharge)										
Death, by cause (%)	9 (0.6)	0 (0)	-	-	1 (0.7)	1.27 (0.15, 11.03)	0.83	5 (2.3)	3.51 (1.04, 11.86)	0.043
Symptomatic recurrent stroke (%)	14 (1.0)	4 (1.8)	1.60 (0.50, 5.09)	0.43	1 (0.7)	0.40 (0.05, 3.29)	0.40	6 (2.8)	2.73 (0.99, 7.52)	0.052
Ischaemic	12 (0.8)	3 (1.3)	1.32 (0.36, 4.90)	0.68	1 (0.7)	0.49 (0.06, 3.92)	0.48	6 (2.8)	3.29 (1.17, 9.26)	0.024
NIH Stroke Scale (/42), calculated	6.8 (5.5)	7.5 (6.1)	0.17 (-0.32, 0.65)	0.50	6.6 (5.4)	-0.17 (-0.77, 0.42)	0.56	9.1 (6.0)	0.86 (0.36, 1.37)	0.001
Clinical deterioration (%)	62 (4.3)	15 (6.7)	1.53 (0.84, 2.77)	0.16	7 (4.8)	0.92 (0.40, 2.10)	0.83	23 (10.8)	2.61 (1.55, 4.39)	<0.001
Neurological deterioration (%)	59 (4.1)	9 (4.0)	0.97 (0.47, 2.02)	0.94	4 (2.7)	0.57 (0.20, 1.63)	0.30	18 (8.5)	2.12 (1.21, 3.74)	0.009
Headache (%)	192 (13.4)	20 (8.9)	0.80 (0.48, 1.32)	0.38	23 (15.5)	1.53 (0.93, 2.51)	0.09	30 (14.1)	1.31 (0.84, 2.03)	0.23
Hypotension (%)	21 (1.5)	3 (1.3)	0.89 (0.25, 3.10)	0.85	3 (2.0)	1.10 (0.31, 3.87)	0.88	2 (0.9)	0.65 (0.15, 2.86)	0.57

Hypertension (%)	78 (5.5)	14 (6.3)	1.22 (0.66, 2.23)	0.53	7 (4.7)	0.97 (0.43, 2.20)	0.94	12 (5.6)	1.06 (0.56, 2.02)	0.86
Death or discharge to institution (%)	356 (24.9)	50 (22.3)	0.81 (0.57, 1.15)	0.24	30 (20.3)	0.61 (0.39, 0.95)	0.029	72 (33.8)	1.34 (0.96, 1.87)	0.08
Day 90										
Barthel Index	77.0 (31.3)	72.8 (35.4)	-1.43 (-5.29, 2.43)	0.47	72.4 (33.6)	-2.44 (-7.10, 2.22)	0.30	61.3 (38.0)	-10.62 (-14.57, -6.67)	<0.001
Zung Depression Scale (ZDS, /100)	52.3 (20.5)	57.2 (22.8)	3.57 (0.55, 6.58)	0.021	53.8 (22.0)	0.65 (-3.06, 4.37)	0.73	61.1 (22.5)	6.53 (3.41, 9.64)	<0.001
EQ-5D Health Utility Status (HUS, /1)	0.57 (0.38)	0.53 (0.37)	-0.01 (-0.06, 0.04)	0.63	0.53 (0.39)	-0.03 (-0.09, 0.03)	0.33	0.40 (0.40)	-0.12 (-0.16, -0.07)	<0.001
EQ-Visual Analogue Scale (EQ-VAS, /100)	63.7 (26.0)	61.4 (29.8)	0.16 (-3.54, 3.87)	0.93	61.2 (28.4)	-1.04 (-5.60, 3.52)	0.66	50.4 (30.1)	-9.58 (-13.42, -5.73)	<0.001
Verbal Fluency	11.7 (7.1)	10.7 (8.3)	-0.32 (-1.56, 0.91)	0.61	10.2 (7.1)	-0.71 (-2.16, 0.75)	0.34	9.6 (7.8)	-0.97 (-2.23, 0.30)	0.14
TICS-M	18.7 (8.8)	16.1 (10.0)	-1.49 (-3.05, 0.07)	0.06	15.9 (9.1)	-1.28 (-3.16, 0.61)	0.18	13.6 (9.7)	-2.92 (-4.55, -1.29)	<0.001
MMSE	14.0 (5.8)	12.0 (6.9)	-1.17 (-2.21, -0.13)	0.028	12.0 (6.5)	-0.97 (-2.24, 0.29)	0.13	10.5 (7.2)	-1.79 (-2.89, -0.69)	0.001

Data are n (%), mean (SD), mean difference (MD) or odds ratio (OR) with 95% confidence intervals. Comparison using logistic or multiple regression with <30% stenosis as reference group. Adjusted for age, sex, baseline mRS, history of previous stroke, history of diabetes mellitus, TACS, nitrate use, baseline SSS, thrombolysis, feeding status, time to randomisation, baseline SBP, GTN/no GTN and continue/stop.

Supplementary table 2: Secondary outcomes by GTN vs. no GTN by degree of ipsilateral carotid stenosis

	Stenosis 30-<50%				Stenosis 50-<70%				Stenosis ≥70%			
	**GTN**	**No GTN**	**OR/MD (95% CI)**	**p**	**GTN**	**No GTN**	**OR/MD (95% CI)**	**p**	**GTN**	**No GTN**	**OR/MD (95% CI)**	**p**
Number of Participants	102	122	-	-	77	71	-	-	94	119	-	-
SBP change day 0-1 (mmHg)*	-13.8 (19.8)	-3.2 (17.9)	-11.0 (-15.7, -6.3)	<0.001	-10.9 (18.1)	-5.6 (15.7)	-3.9 (-9.6, 18)	0.18	-8.9 (17.5)	-3.1 (17.1)	-6.5 (-11.0, -2.0)	0.005
Day 7 (or discharge)												
Death, by cause (%)	0 (0)	0 (0)	-	-	1 (1.3)	0 (0)	-	-	3 (3.2)	2 (1.7)	-	-
Symptomatic recurrent stroke (%)	1 (1.0)	3 (2.5)	0.09 (0.00, 11.08)	0.33	0 (0)	1 (1.4)	-	-	3 (3.2)	3 (2.5)	0.87 (0.09, 8.21)	0.90
Ischaemic	1 (1.0)	2 (1.6)	0.18 (0.00, 11.17)	0.41	0 (0)	1 (1.4)	-	-	3 (3.2)	3 (2.5)	0.87 (0.09, 8.21)	0.90
NIH Stroke Scale (/42), calculated	7.3 (6.2)	7.6 (6.0)	-0.41 (-1.46, 0.64)	0.44	6.6 (5.0)	6.6 (5.8)	-0.75 (-1.96, 0.46)	0.22	8.9 (6.3)	9.2 (5.8)	-0.55 (-1.75, 0.66)	0.37
Clinical deterioration (%)	6 (5.9)	9 (7.4)	0.69 (0.19, 2.49)	0.57	3 (3.9)	4 (5.6)	0.53 (0.06, 4.46)	0.56	10 (10.6)	13 (11.0)	0.89 (0.35, 2.27)	0.89
Neurological deterioration (%)	3 (2.9)	6 (4.9)	0.23 (0.03, 2.12)	0.20	1 (1.3)	3 (4.2)	-	-	11 (11.7)	7 (5.9)	2.33 (0.79, 6.83)	0.13
Headache (%)	12 (11.8)	8 (6.6)	2.03 (0.70, 5.94)	0.19	16 (20.8)	7 (9.9)	2.73 (0.96, 7.72)	0.059	19 (20.2)	11 (9.2)	2.80 (1.18, 6.65)	0.019
Hypotension (%)	3 (2.9)	0 (0)	-	-	3 (3.9)	0 (0)	-	-	2 (2.1)	0 (0)	-	-
Hypertension (%)	6 (5.9)	8 (6.6)	0.93 (0.26, 3.40)	0.91	3 (3.9)	4 (5.6)	162.4 (0.03, 925848)	0.25	5 (5.3)	7 (5.9)	0.76 (0.21, 2.82)	0.68

Death or discharge to institution (%)	22 (21.6)	28 (23.0)	0.84 (0.41, 1.75)	0.65	13 (16.9)	17 (23.9)	0.64 (0.25, 1.62)	0.34	27 (28.7)	45 (37.8)	0.51 (0.26, 1.00)	0.050
Day 90												
Barthel Index	75.2 (34.4)	70.8 (36.2)	5.78 (-2.37, 13.94)	0.16	74.9 (31.5)	69.7 (35.8)	7.86 (-2.24, 17.95)	0.13	65.7 (38.0)	57.9 (37.9)	7.87 (-0.10, 16.74)	0.08
Zung Depression Scale (ZDS, /100)	56.5 (22.2)	57.8 (23.3)	-0.89 (-7.20, 5.43)	0.78	51.1 (19.2)	56.8 (24.6)	-7.33 (-14.93, 0.27)	0.059	59.7 (22.6)	62.1 (22.6)	-3.78 (-9.87, 2.32)	0.22
EQ-5D Health Utility Status (HUS, /1)	0.54 (0.38)	0.53 (0.36)	0.00 (-0.08, 0.09)	0.94	0.53 (0.39)	0.53 (0.40)	0.05 (-0.07, 0.17)	0.44	0.44 (0.39)	0.37 (0.40)	0.07 (-0.02, 0.17)	0.11
EQ-Visual Analogue Scale (EQ-VAS, /100)	62.4 (28.6)	60.5 (30.8)	2.12 (-5.81, 10.05)	0.60	62.6 (26.0)	59.7 (30.9)	5.57 (-3.96, 15.10)	0.25	54.3 (29.6)	47.4 (30.4)	6.91 (-1.04, 14.85)	0.09
Verbal Fluency	11.3 (8.1)	10.2 (8.5)	0.78 (-1.97, 3.52)	0.58	10.6 (6.5)	9.8 (7.6)	0.96 (-1.54, 3.47)	0.45	9.5 (7.0)	9.7 (8.5)	0.23 (-2.48, 2.94)	0.87
TICS-M	15.8 (9.2)	16.3 (10.6)	-0.88 (-4.16, 2.41)	0.60	18.4 (7.5)	13.3 (10.0)	4.92 (1.36, 8.49)	0.008	14.7 (9.8)	12.8 (9.6)	2.34 (-1.20, 5.88)	0.19
MMSE	12.2 (6.4)	11.9 (7.2)	0.26 (-1.94, 2.45)	0.82	14.0 (5.2)	10.1 (7.1)	4.11 (1.63, 6.59)	0.002	11.3 (4.0)	10.1 (7.4)	1.34 (-1.25, 3.94)	0.31

Data are n (%), mean (SD), mean difference (MD) or odds ratio (OR) with 95% confidence intervals. Comparison using logistic or multiple regression with <30% stenosis as reference group. Adjusted for age, sex, baseline mRS, history of previous stroke, history of diabetes mellitus, TACS, nitrate use, baseline SSS, thrombolysis, feeding status, time to randomisation, baseline SBP and continue/stop. *=adjusted for baseline SBP only

Supplementary table 3: Secondary outcomes by stop vs. continue antihypertensives by degree of ipsilateral carotid stenosis

	Stenosis 30-<50%				Stenosis 50-<70%				Stenosis ≥70%			
	**Cont**	**Stop**	**OR/MD (95% CI)**	**p**	**Cont**	**Stop**	**OR/MD (95% CI)**	**p**	**Cont**	**Stop**	**OR/MD (95% CI)**	**p**
Number of Participants	65	68	-	-	52	47	-	-	57	57	-	-
SBP change day 0-1 (mmHg)*	-8.7 (17.3)	-6.0 (21.0)	-3.48 (-9.91, 2.96)	0.29	-8.4 (17.3)	-6.0 (18.0)	-4.12 (-11.39, 3.15)	0.26	-5.8 (16.6)	-6.4 (14.6)	0.01 (-5.76, 5.77)	0.99
Day 7 (or discharge)												
Death, by cause (%)	0 (0)	0 (0)	-	-	1 (1.9)	0 (0)	-	-	1 (1.8)	1 (1.8)	-	-
Symptomatic recurrent stroke (%)	2 (3.1)	2 (2.9)	1.05 (0.05, 22.98)	0.97	0 (0)	1 (2.1)	-	-	2 (3.5)	2 (3.5)	-	-
Ischaemic	2 (3.1)	1 (1.5)	5.13 (0.08, 338.19)	0.44	0 (0)	1 (2.1)	-	-	2 (3.5)	2 (3.5)	-	-
NIH Stroke Scale (/42), calculated	7.6 (6.4)	8.0 (6.6)	0.63 (-0.98, 2.23)	0.44	7.3 (5.7)	6.7 (4.8)	0.45 (-1.17, 2.07)	0.58	8.6 (6.2)	9.2 (5.5)	-0.66 (-2.30, 0.97)	0.42
Clinical deterioration (%)	6 (9.2)	5 (7.5)	1.95 (0.40, 9.51)	0.41	5 (9.8)	1 (2.1)	10.60 (0.63, 177.41)	0.10	6 (10.7)	5 (8.8)	2.05 (0.38, 11.08)	0.40
Neurological deterioration (%)	4 (6.2)	2 (2.9)	-	-	2 (3.8)	0 (0)	-	-	4 (7.0)	2 (3.5)	2.00 (0.19, 21.11)	0.57
Headache (%)	4 (6.2)	7 (10.3)	0.53 (0.10, 2.77)	0.45	9 (17.3)	7 (14.9)	1.03 (0.29, 3.63)	0.96	7 (12.3)	8 (14.0)	1.04 (0.20, 5.41)	0.96
Hypotension (%)	1 (1.5)	1 (1.5)	-	-	0 (0)	3 (6.4)	-	-	0 (0)	0 (0)	-	-
Hypertension (%)	2 (3.1)	6 (8.8)	0.38 (0.04, 3.35)	0.38	1 (1.9)	5 (10.6)	-	-	1 (1.8)	6 (10.5)	0.09 (0.01, 1.34)	0.08

Death or discharge to institution (%)	17 (26.2)	17 (25.0)	1.37 (0.51, 3.65)	0.53	10 (19.2)	8 (6.5)	2.47 (0.66, 9.18)	0.18	21 (36.8)	16 (28.1)	1.39 (0.54, 3.61)	0.50
Day 90												
Barthel Index	70.2 (38.3)	71.3 (36.3)	-2.48 (-14.27, 9.32)	0.68	67.5 (36.1)	74.9 (27.1)	-13.20 (-26.29, -0.11)	0.048	58.7 (37.8)	50.3 (42.5)	10.73 (-24.40, 2.94)	0.12
Zung Depression Scale (ZDS, /100)	61.2 (24.6)	60.0 (22.1)	1.20 (-7.55, 9.96)	0.79	60.9 (23.3)	48.8 (17.8)	13.18 (3.80, 22.56)	0.007	67.6 (23.9)	61.6 (22.4)	10.21 (1.10, 19.33)	0.029
EQ-5D Health Utility Status (HUS, /1)	0.49 (0.41)	0.51 (0.36)	-0.03 (-0.15, 0.09)	0.62	0.48 (0.39)	0.56 (0.33)	-0.16 (-0.30, -0.01)	0.036	0.33 (0.43)	0.35 (0.39)	-0.06 (-0.20, 0.07)	0.37
EQ-Visual Analogue Scale (EQ-VAS, /100)	57.0 (31.5)	59.5 (29.3)	-2.04 (-12.69, 8.62)	0.71	54.0 (32.3)	68.1 (20.4)	-17.20 (-29.41, -4.99)	0.006	44.1 (33.8)	48.3 (27.0)	-6.22 (-18.56, 6.13)	0.32
Verbal Fluency	8.5 (7.5)	10.5 (7.7)	-2.08 (-5.71, 1.55)	0.26	7.6 (6.0)	11.3 (5.0)	-2.69 (-5.61, 0.22)	0.07	6.7 (6.2)	9.7 (8.6)	-3.11 (-6.84, 0.62)	0.10
TICS-M	12.9 (9.4)	15.8 (10.0)	-2.38 (-7.28, 2.52)	0.34	13.2 (9.9)	19.3 (7.8)	-5.66 (-10.40, -0.92)	0.021	11.1 (10.2)	13.1 (9.3)	-3.39 (-8.45, 1.67)	0.18
MMSE	10.1 (7.1)	11.7 (6.9)	-1.30 (-4.76, 2.16)	0.45	10.1 (7.4)	13.9 (5.1)	-2.71 (-6.57, 1.15)	0.16	8.3 (7.8)	10.4 (6.7)	-2.80 (-6.52, 0.92)	0.14

Data are n (%), mean (SD), mean difference (MD) or odds ratio (OR) with 95% confidence intervals. Comparison using logistic or multiple regression. Adjusted for age, sex, baseline mRS, history of previous stroke, history of diabetes mellitus, TACS, nitrate use, baseline SSS, thrombolysis, feeding status, time to randomisation, baseline SBP and GTN/no GTN.
*=adjusted for baseline SBP only

Supplementary table 4: Outcomes by degree of bilateral carotid stenosis

	Stenosis <30%	Stenosis 30-<50%			Stenosis ≥50%		
		n (%) / mean (SD)	OR/MD (95% CI)	p	n (%) / mean (SD)	OR/MD (95% CI)	p
Number of Participants	1287	115	-	-	97	-	-
Primary outcome	1282	115	-	-	96	-	-
mRS (/6)*	2 [3]	2 [3]	0.91 (0.65, 1.29)	0.61	3 [2]	0.99 (0.60, 1.61)	0.95
Secondary outcomes							
Day 7 (or Discharge)							
Death, by cause (%)	9 (0.7)	0 (0)	-	-	1 (1.0)	1.72 (0.18, 16.35)	0.64
Symptomatic recurrent stroke (%)	10 (0.8)	2 (1.7)	1.85 (0.38, 9.04)	0.45	1 (1.0)	0.89 (0.10, 7.84)	0.91
Ischaemic	9 (0.7)	1 (0.9)	1.08 (0.13, 9.13)	0.95	1 (1.0)	0.86 (0.09, 8.19)	0.90
NIH Stroke Scale (/42), calculated	6.8 (5.4)	6.9 (6.3)	-0.06 (-0.69, 0.58)	0.85	6.8 (5.1)	-0.15 (-0.84, 0.55)	0.68
Clinical deterioration (%)	58 (4.5)	9 (7.9)	1.54 (0.72, 3.28)	0.27	5 (5.2)	0.95 (0.36, 2.53)	0.91
Neurological deterioration (%)	52 (4.0)	6 (5.2)	1.20 (0.49, 2.92)	0.69	2 (2.1)	0.47 (0.11, 1.98)	0.30
Headache (%)	179 (13.9)	11 (9.6)	0.82 (0.42, 1.61)	0.57	11 (11.3)	0.95 (0.48, 1.87)	0.88
Hypotension (%)	20 (1.6)	1 (0.9)	0.52 (0.07, 4.10)	0.53	2 (2.1)	1.06 (0.23, 4.85)	0.94
Hypertension (%)	73 (5.7)	8 (7.0)	1.32 (0.61, 2.88)	0.49	6 (6.2)	1.19 (0.48, 2.95)	0.70

Death or discharge to institution (%)	327 (25.4)	28 (24.3)	0.85 (0.53, 1.37)	0.50	24 (24.7)	0.80 (0.48, 1.34)	0.40
Day 90							
Death (%)	62 (4.8)	11 (9.6)	1.94 (0.93, 4.06)	0.08	7 (7.3)	1.47 (0.61, 3.52)	0.39
Barthel Index	76.9 (31.6)	75.0 (35.8)	0.65 (-4.41, 5.71)	0.80	70.8 (33.7)	-3.78 (-9.32, 1.77)	0.18
Zung Depression Scale (ZDS, /100)	52.4 (20.7)	57.2 (23.1)	3.91 (-0.10, 7.92)	0.056	55.6 (20.8)	2.56 (-1.80, 6.92)	0.25
EQ-5D Health Utility Status (HUS, /1)	0.56 (0.38)	0.56 (0.36)	0.01 (-0.05, 0.07)	0.74	0.51 (0.37)	-0.04 (-0.11, 0.03)	0.23
EQ-Visual Analogue Scale (EQ-VAS, /100)	63.4 (26.2)	63.4 (30.2)	1.76 (-3.14, 6.66)	0.48	59.0 (28.3)	-3.42 (-9.02, 2.18)	0.23
Verbal Fluency	11.8 (7.2)	11.2 (8.0)	0.18 (-1.39, 1.75)	0.82	10.6 (7.6)	-0.68 (-2.44, 1.09)	0.45
TICS-M	18.7 (8.7)	16.7 (9.6)	-0.68 (-2.61, 1.26)	0.49	15.8 (8.4)	-1.96 (-4.16, 0.24)	0.08
MMSE	13.9 (5.8)	12.5 (6.5)	-0.44 (-1.72, 0.84)	0.50	12.6 (6.3)	-0.77 (-2.25, 0.70)	0.30

Data are n (%), mean (SD), median [interquartile range], mean difference (MD) or odds ratio (OR) with 95% confidence intervals. Comparison using logistic regression, multiple regression or ordinal regression. Adjusted for age, sex, baseline mRS, history of previous stroke, history of diabetes mellitus, TACS, nitrate use, baseline SSS, thrombolysis, feeding status, time to randomisation, baseline SBP, GTN/no GTN and continue/stop. *=ordinal logistic regression.

Supplementary table 5: Change in BP from baseline to day 1 and association with mRS at day 90 by degree of ipsilateral carotid stenosis and treatment allocation

Carotid stenosis (%)	Overall			GTN vs. no GTN			Stop vs. Continue		
	ΔSBP	OR (95% CI)	p	Interaction p	OR (95% CI)	p	Interaction p	OR (95% CI)	p
<30	-8.0 (18.7)	0.95 (0.90, 1.00)	0.07	0.37	1.05 (0.87, 1.28)	0.61	0.88	0.96 (0.73, 1.26)	0.77
30-50	-8.1 (19.5)	0.93 (0.81, 1.07)	0.31	0.63	0.84 (0.50, 1.42)	0.52	0.94	0.91 (0.46, 1.80)	0.79
50-70	-8.3 (17.1)	0.80 (0.66, 0.97)	0.026	0.66	0.77 (0.41, 1.45)	0.41	0.44	1.93 (0.85, 4.41)	0.12
≥70	-5.7 (17.5)	1.05 (0.90, 1.22)	0.55	0.34	0.53 (0.32, 0.90)	0.019	0.98	1.44 (0.72, 2.91)	0.31

ΔSBP = SBP day 0 - SBP day 1 (mmHg). Data are mean (SD) or odds ratio (OR) with 95% confidence intervals. Comparison using ordinal regression. Adjusted for age, sex, baseline mRS, history of previous stroke, history of diabetes mellitus, TACS, nitrate use, baseline SSS, thrombolysis, feeding status, time to randomisation, baseline SBP, stop/continue antihypertensives and GTN/no GTN. Overall ORs per 10mmHg reduction in BP.

Supplementary table 6: Change in BP from baseline to day 1 and association with mRS at day 90 by degree of bilateral carotid stenosis and treatment allocation

Carotid stenosis (%)	Overall			GTN vs. no GTN			Stop vs. Continue		
	ΔSBP	OR (95% CI)	p	Interaction p	OR (95% CI)	p	Interaction p	OR (95% CI)	p
<30	-8.1 (18.4)	0.95 (0.90, 1.01)	0.09	0.10	1.02 (0.83, 1.26)	0.83	0.66	0.94 (0.70, 1.25)	0.66
30-50	-5.6 (19.4)	0.83 (0.67, 1.03)	0.09	0.22	1.27 (0.57, 2.83)	0.56	0.22	1.05 (0.37, 3.01)	0.92
≥50	-8.6 (19.5)	1.95 (0.74, 1.21)	0.65	0.21	1.01 (0.44, 2.32)	0.99	0.59	0.54 (0.16, 1.86)	0.33

ΔSBP = SBP day 0 - SBP day 1 (mmHg). Data are n (%), mean (SD), median [interquartile range], mean difference (MD) or odds ratio (OR) with 95% confidence intervals. Comparison using ordinal regression. Adjusted for age, sex, baseline mRS, history of previous stroke, history of diabetes mellitus, TACS, nitrate use, baseline SSS, thrombolysis, feeding status, time to randomisation, baseline SBP, stop/continue antihypertensives and GTN/no GTN. Overall ORs per 10mmHg reduction in BP.
